# Deep medullary veins: a promising neuroimaging marker for mild cognitive impairment in outpatients

**DOI:** 10.1186/s12883-022-03037-x

**Published:** 2023-01-05

**Authors:** Xiuqi Chen, Yufan Luo, Shufan Zhang, Xiaoli Yang, Zhiyuan Dong, Yilin Wang, Danhong Wu

**Affiliations:** 1 Department of Neurology, Shanghai Fifth People’s Hospital, Fudan University, No.801, He Qing Road, Minhang District, Shanghai, China; 2 Department of Neurology, Shanghai Jing’an District Central Hospital, Fudan University, Shanghai, China; 3 Georgetown Preparatory School, North Bethesda, MD Washington, USA

**Keywords:** Mild cognitive impairment, Deep medullary vein, Neuropsychologic test

## Abstract

**Background and purpose:**

Mild cognitive impairment is an age-dependent pre-dementia state caused by varied reasons. Early detection of MCI helps handle dementia. Vascular factors are vital for the occurrence of MCI. This study investigates the correlation between deep medullary veins and multi-dimensional cognitive outcomes.

**Materials and methods:**

A total of 73 participants with MCI and 32 controls were enrolled. Minimum Mental State Examination and Montreal Cognitive Assessment were used to examine the global cognitive function, and different cognitive domains were measured by specific neuropsychological tests. MRI was used to assess the visibility of the DMV and other neuroimage markers.

**Results:**

DMV score was statistically significantly higher in the MCI group compared with the control group (*P* = 0.009) and independently related to MCI (*P* = 0.007). Linear regression analysis verified that DMV score was linearly related to global cognition, memory, attention, and executive function after adjusting for cerebrovascular risk factors.

**Conclusion:**

DMV score was independently related to the onset of MCI, and correlates with overall cognition, memory, attention, and executive function in outpatients.

**Supplementary Information:**

The online version contains supplementary material available at 10.1186/s12883-022-03037-x.

## Introduction

As the lifespan of people extends, cognitive impairment arises as a vital issue. Alzheimer’s disease (AD) is known to be the most common cause of cognitive impairment, with the risk doubling as age grows every 4.5 years (O’Brien & Thomas, [29]), and cognitive dysfunction caused by vascular factors ranked the second reason with the risk doubling as age grows every 5.3 years (Gorelick et al., [12]; O’Brien & Thomas, [29]). There is a paucity of efficient therapeutic approaches for dementia (O’Brien & Thomas, [29]), but early detection and intervention of cognitive decline in the elderly may benefit. Mild cognitive impairment (MCI) depicts a clinical situation that personal autonomy is preserved in patients with clinically recognizable cognitive decline (Gauthier et al., [11]). The cognitive status of patients with MCI is damaged but does not reach the extent of dementia, which indicates that early detection of MCI is a vital strategy for rivaling dementia.

In clinics, people are more likely to suffer from MCI of mixed causes (O’Brien & Thomas, [29]), and cerebral small vessel disease (CSVD) is the main reason for cognitive impairment attributed to vascular factors (Casado, Encarnación López-Fernández, Concepción Casado, & de La Torre, [3]). Previous research has discovered that white matter hyperintensity (WMH), lacunar infarcts (LI), cerebral microbleeds (CMB), and enlarged perivascular space (EPVS) in CSVD participate in cognitive impairment onset (Gouveia-Freitas & Bastos-Leite, [13]; Han et al. [18]; Pantoni, [30]; Sepehrband et al., [34]; Wang et al., [37]; Williamson et al., [38]; Yakushiji et al., [39]). and these pathological lesions can be caused by the changes in the cerebral small venous system via aging-dependent venous wall collagen deposit and remodeling (Molnár et al., [26]). It is reasonable to postulate that alternations of venules may become indicators of cognitive impairment. Deep medullary veins (DMV) run in the periventricular area to collect venous blood and return it to the heart. Since SWI can distinguish DMV from surrounding brain tissue and arterioles by detecting paramagnetic deoxyhemoglobin in venous blood (Reichenbach, Venkatesan, Schillinger, Kido, & Haacke, [32]), DMV may serve as a window for observing the pathological alternations of cerebral veins.

Our study aimed to assess the relationship between DMV and overall cognitive impairment as well as multiple cognitive domains in outpatients. In the future, DMV score might be the potential neuroimaging biomarker for MCI.

## Materials and methods

### Participants

**Fig. 1:**
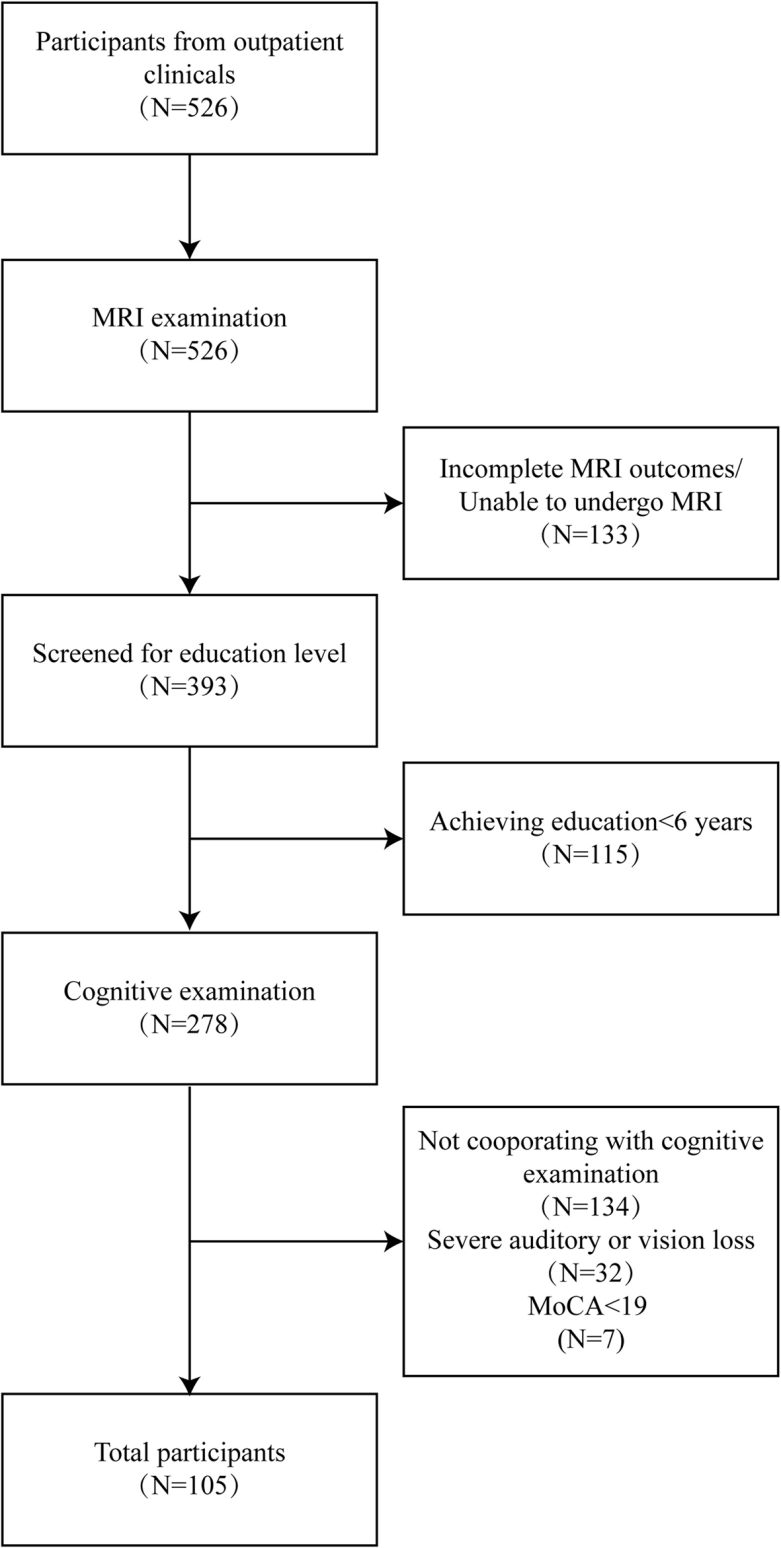
Flow chart

Five hundred and twenty-six participants from outpatient clinical were consecutively screened from June 2017 to September 2020. One hundred and five participants meeting the following inclusion criteria were selected: (1) ≥ 40 years old, (2) achieved education higher than 6 years, (3) had no history of cerebrovascular accidents, (4) had no physical severe disease (e.g. myocardial infarction or heart failure), (5) had no visual or auditory deficit, (6) had no contraindication for MRI. The detailed screening process is shown in Fig. 1.

### Vascular risk factors

Neurologists collected demographic and clinical information for all participants, including sex, age, hypertension, diabetes, history of smoking, history of drinking, education, total cholesterol (TC), triglyceride (TG), high-density lipoprotein (HDL), low-density lipoprotein (LDL), fasting blood glucose (FBG) and glycated hemoglobin glycosylated hemoglobin (HbA1c).

### MRI examination

MRI was used to assess the visibility of DMV. All participants underwent a whole-brain MRI. MRI in this paper was acquired using a 3.0 T MRI scanner (Signa HDxt; GE HealthCare, Milwaukee, WI, USA).

### Detection and evaluation of DMV on MRI

DMV evaluation was conducted on the SWI sequence. This study has assessed 6 segments (bilateral frontal lobes, parietal lobes, and occipital lobes), and DMV was scaled as 0 (consecutive veins and even signals), 1 (consecutive veins but one or more veins present uneven signals), 2 (one or more discontinuous veins and dotted hypointensity) or 3 (no visible consecutive vein) (Mucke et al., [28]) (Supplementary Fig. 1). The total DMV score was counted as the sum of 6 segments. Higher scores indicate worse visibility of DMV. The DMV assessments were independently evaluated by two neurologists, and the inter-observer agreement value for DMV is 0.82. Any disagreement was resolved by consensus with the third neuroimaging expert.

### Detection and evaluation of CSVD lesions on MRI

Neuroimaging lesions of CSVD include WMH, LI, CMB, and EPVS according to the STRIVE recommendation.

#### WMH

WMH was defined as abnormal hyperintensity on T2 and Flair in periventricular or deep white matter parenchyma. WMH grading conforms to Fazekas score criteria. Higher scores represent more severe WMH. That deep white matter hyperintensity (DWMH) score or periventricular hyperintensity (PVH) score did not equal zero was defined as WMH presence, and DWMH score ≥ 2 or PVH score = 3 was defined as severe WMH (Fazekas, Chawluk, Alavi, Hurtig, & Zimmerman, [9]).

#### LI

LI was defined as round or oval lesions with a diameter of 3-15 mm and located in the basal ganglia, thalamus, internal capsule, external capsule, or brain stem. It presents inner hypointensity with surrounded hyperintensity in Flair sequence (Klarenbeek, van Oostenbrugge, Rouhl, Knottnerus, & Staals, [22]).

#### CMB

CMB was defined as hypointensity lesions with a diameter < 10 mm in SWI sequence (Greenberg et al., [14]).

#### EPVS

EPVS was defined as round or oval lesions with a diameter < 2 mm in basal ganglia and centrum semiovale. It presents hypointensity in Flair sequence (Klarenbeek, van Oostenbrugge, Lodder, et al., [21]). EPVS was counted as grade 1 (0–10 EPVS lesions); grade 2 (11–25 EPVS lesions) and grade 3 (> 25 EPVS lesions) (Greenberg et al., [14]; Klarenbeek, van Oostenbrugge, Lodder, et al., [21]).

The assessments for CSVD lesions were also independently evaluated by two neurologists, and the inter-observer agreement value for DWMH, PVH, LI, CMB, EPVS in basal ganglia and EPVS in centrum semiovale were 0.78, 0.80, 0.82, 0.83 and 0.79 respectively. Any disagreement was resolved by consensus with the third neuroimaging expert.

### Cognitive assessment

Participants with Montreal Cognitive Assessment (MoCA) score of 19 to 25 were defined as MCI (de Havenon et al. [5]). Minimum Mental State Examination (MMSE) was used to assist in evaluating global cognition. Different cognitive domains were evaluated by specific neuropsychological tests. All tests were finished by at least two neurologists.

#### AVLT

AVLT (Auditory verbal learning test) assessed the memory of participants. The inspector read words (3 categories and 4 words each category) to participants and participants began to recall as soon as the inspector had finished. This process was repeated 3 times and the number of correct recollections were named as N1, N2, and N3, respectively. After the five-minute non-verbal test, the participants were required to recall the words the fourth time (N4) and repeat this step the fifth time (N5) after another twenty-minute non-verbal test. The memory of participants was measured according to the following criteria(Guo, Zhao, Chen, Ding, & Hong, [15]): (1) short-term memory (N1 + N2 + N3); (2) short-time delayed recall (N4) and (3) long-time delayed recall (N5).

#### SDMT

SDMT (Symbol digit modalities test) assessed the attention of participants. Participants were required to memorize digits (1–9) and their symbol’s correspondence in the test table. In the formal test, participants wrote down digits corresponding to symbols. The number of right items in 90 s was recorded as the final score (Pantoni et al., [31]).

#### TMT

TMT (Trail making test) assessed the attention of participants. It was composed of two tests. In test A, participants were told to connect in order a sequence of 25 digits (1–25); ln test B, participants were told to connect a sequence of 25 digits in order and the frame of adjacent digits should not be the same. Total seconds of test B was recorded as the final score (Du et al., [8]).

#### SCWT

SCWT (Stroop color and word test) assessed the executive function of participants. It includes three tests. In test A, participants were required to read the characters on the card in order; In test B, participants were required to read the color of ovals on the card in order; ln test C, participants were required to read the color of the characters in order. The total seconds of test C were recorded as the final score (Camerino et al., [2]).

#### VFT

VFT assessed the verbal function of participants. Participants were required to enumerate as many as possible animals in 60 s, and the total number was recorded as the final score (Zhao, Guo, & Hong, [40]).

### Statistical analysis

All statistical analyses were performed on SPSS (version 25.0). Continuous data conforming to normal distribution was described by mean (standard deviation), otherwise, it was depicted by median [low quartile (Q_25_), upper quartile (Q_75_)]. T-test or Wilcoxon test was selected for comparing continuous data of two groups, Chi-square test or Fisher’s exact test was chosen for comparing dichotomous variable of two groups. Binary logistic regression was used to analyze the relation between DMV score and MCI, and the variables with the *P* value < 0.1 in univariate regression were included in the multivariate regression model. Stepwise multiple linear regression analysis was used to assess the relationship between DMV scores and various cognitive domains and bidirectional elimination was used to filter factors. A two-tailed *P* value of < 0.1 was taken as the entry criteria, and a two-tailed *P* value of < 0.05 was taken as the exclusion criteria. Spearman’s correlation was used to assess the associations of the DMV score as well as other neuroimaging markers with the MMSE, MoCA, and outcomes of different cognitive domains. A two-tailed *P* value of < 0.05 was considered statistically significant.

## Results

### Demographics

This research enrolled 105 eligible participants in total. The median age of the included patients was 68.0 (Q_25_, Q_75_= [63.5, 73.0]) years, and 65.7% of them were female (*N* = 69). Seventy-three participants were diagnosed with MCI. Sex (*P* = 0.026), DMV score (*P* = 0.009), education level (*P* = 0.003), MMSE (*P* = 0.002), and MoCA (*P* < 0.001) exhibited a statistically significant difference between the MCI group and control group. Detailed information was listed in Table 1. We also compared neuroimaging markers for small vessel disease (Supplementary Table 1), but no statistical difference was found between the MCI group and the control group.

**Table 1:** Baseline characteristics

Characteristics	Total (*N* = 105)	MCI group (*N* = 73)	Control group (*N* = 32)	*P*
Sex				
Male, n,(%)	36 (34.3)	30 (41.1)	6 (18.8)	0.026*
Female, n,(%)	69 (65.7)	43 (58.9)	26 (81.2)	
Age, years, median(Q25, Q75)	68.0 (63.5, 73.0)	67.0 (63.0, 73.0)	68.5 (65.0, 72.8)	0.638
Smoking, n,(%)	27 (25.7)	24 (32.9)	3 (9.4)	0.110
Drinking, n,(%)	32 (30.5)	25 (34.2)	7 (21.8)	0.205
Hypertension, n,(%)	61 (58.1)	45 (61.6)	16 (50.0)	0.266
Diabetes, n,(%)	23 (21.9)	14 (19.2)	9 (28.1)	0.308
TC,mmol·L-1, median(Q25, Q75)	4.55 (3.77, 5.10)	4.57 (3.71, 5.23)	4.46 (4.02, 4.95)	0.914
TG,mmol·L-1, median(Q25, Q75)	1.33 (1.01, 1.69)	1.56 (1.26, 1.72)	1.32 (0.88, 1.76)	0.978
HDL, mmol·L-1, mean(SD)	1.42 (0.37)	1.38 (0.37)	1.52 (0.35)	0.076#
LDL, mmol·L-1, median(Q25, Q75)	2.90 (2.32, 3.45)	2.88 (2.13, 3.43)	2.96 (2.41, 3.52)	0.493
FBG, mmol·L-1, median(Q25, Q75)	5.27 (4.83, 5.81)	5.27 (4.81, 5.86)	5.25 (4.83, 5.56)	0.876
HbA1c, mmol·L-1, median(Q25, Q75)	5.60 (5.30, 6.05)	5.70 (5.30, 6.10)	5.55 (5.21, 5.98)	0.101
DMV score, mean(SD)	7.95 (5.18)	8.82 (5.25)	5.97 (4.52)	0.009*
Education level,n,(%)				0.003*
< 16 years	92 (87.6)	69 (94.5)	23 (71.9)	
≥ 16 years	13 (12.4)	4 (5.5)	9 (28.1)	
**MMSE, median(Q25, Q75)**	28.0 (27.0, 29.0)	28.0 (27.0, 29.0)	29.0 (28.0, 30.0)	0.002*
MoCA, mean(SD)	23.97 (2.76)	22.52 (1.89)	27.28 (1.11)	< 0.001*

^*^ *P* < 0.05

### Logistic regression analysis

After adjusting for sex, smoking and HDL, HbA1c (OR = 2.507, 95%CI=[1.101, 5.708], *P* = 0.029), DMV score (OR = 1.162, 95%CI=(1.043, 1.295), *P* = 0.007) and education level (OR = 0.158, 95% CI=(0.037, 0.672), *P* = 0.012) were independently related to MCI. More details were listed in Table 2.

**Table 2:** Binary logistic regression analysis for the association between DMV and MCI

Characteristics	Univariate regression		Multivariate regression	
	OR (95% CI)	*P*	OR (95% CI)	*P*
Male	3.023 (1.109, 8.240)	0.031*	1.966 (0.457, 8.467)	0.364
Age	0.993 (0.929, 1.061)	0.829		
Smoking	4.735 (1.310, 17.116)	0.018*	4.301 (0.658, 28.118)	0.128
Drinking	1.860 (0.707, 4.895)	0.209		
Hypertension	1.607 (0.695, 3.716)	0.267		
Diabetes	0.606 (0.231, 1.593)	0.310		
TC	1.025 (0.720, 1.459)	0.892		
TG	0.910 (0.565, 1.466)	0.698		
HDL	0.351 (0.110, 1.128)	0.078#	0.407 (0.101, 1.646)	0.207
LDL	1.029 (0.875, 1.211)	0.729		
FBG	1.194 (0.746, 1.912)	0.461		
HbA1c	2.031 (1.014, 4.067)	0.045*	2.507 (1.101, 5.708)	0.029*
DMV score	1.123 (1.027. 1.229)	0.011*	1.162 (1.043, 1.295)	0.007*
Education level				
< 16 years	Reference			
≥ 16 years	0.148 (0.042, 0.527)	0.003*	0.158 (0.037, 0.672)	0.012*

^*^ *P* < 0.05; # 0.05 < *P* < 0.1

### Correlation analysis

**Fig. 2:**
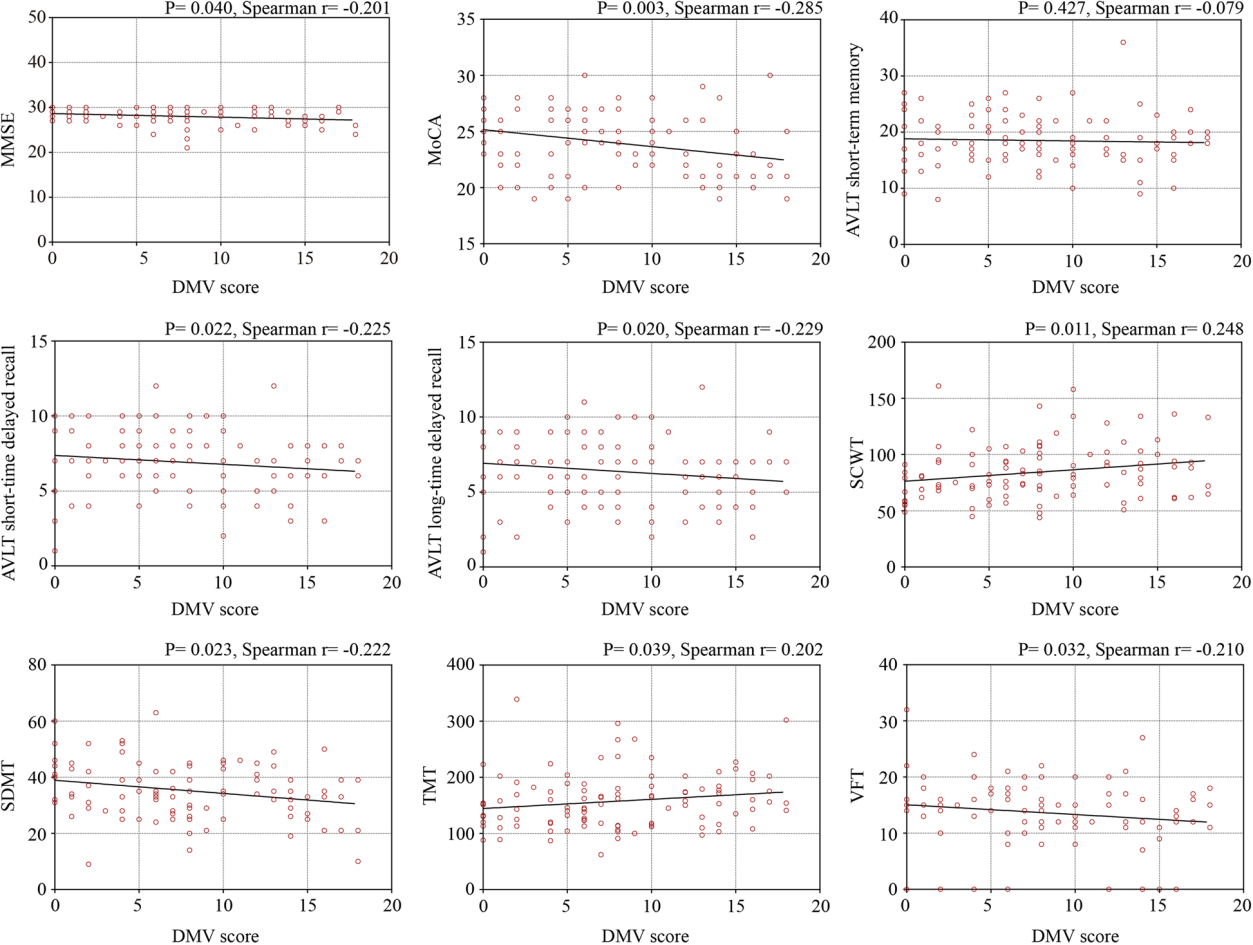
Spearman’s correlation between DMV score and cognition

Spearman’s correlation analysis showed a significant correlation between the DMV score and the MMSE (*P* = 0.040), MoCA (*P* = 0.003), AVLT short-term delay (*P* = 0.022), AVLT long-term delay (*P* = 0.020), SDMT (*P* = 0.023), SCWT (*P* = 0.011), TMT (*P *= 0.039) and VFT (*P* = 0.032). (Fig. 2). This study also performed Spearman’s correlation analysis on the relationship between neuroimaging markers for small vessel disease (including DWMH, PVH, EPVS in basal ganglia, EPVS in centrum semiovale, CMB and LI) and cognitive domains, and only DWMH reveals a significant correlation with VFT (*P* = 0.032). (Supplementary Fig. 2)

### Multivariate linear regression analysis of DMV score and cognition

Stepwise linear regression analysis was used to detect the relationship between DMV score and overall cognition and different cognitive domains. After adjusting for confounding factors, DMV score had a negative association with MMSE(Standardized β coefficient=-0.244, 95%CI= (-0.433, -0.054), *P* = 0.012), MoCA (Standardized β coefficient=-0.235, 95%CI= (-0.412, -0.058), *P* = 0.010), AVLT short-time delayed recall (Standardized β coefficient=-0.198, 95%CI= (-0.388, -0.010), *P* = 0.040), AVLT long-time delayed recall (Standardized β coefficient=-0.206, 95%CI= (-0.396, -0.016), *P* = 0.034), SDMT (Standardized β coefficient=-0.233, 95%CI= (-0.413, -0.053), *P* = 0.012) and had a positive association with SCWT (Standardized β coefficient = 0.251, 95%CI= (0.073, 0.429), *P* = 0.006). (Table 3)

**Table 3:** Stepwise multiple linear regression analysis for the association between DMV score and cognition

Characteristics	Unadjusted		Multivariable regression analysis	
	Unstandardized β coefficient (95% CI)	*P*	Standardized β coefficient (95% CI)	*P*
**MMSE**	**-0.081 (-0.144, -0.018)**	**0.012**	**-0.244 (-0.433, -0.052)**	0.012*
MoCA	-0.150 (-0251,-0.050)	0.004*	-0.235 (-0.412, -0.058)	0.010*
AVLT short-term memory	-0.067 (-0.240, 0.105)	0.441		
AVLT short-time delayed recall	-0.078 (-0.152, -0.004)	0.039*	-0.198 (-0.388, -0.010)	0.040*
AVLT long-time delayed recall	-0.084 (-0.168, -0.001)	0.046*	-0.206 (-0.396, -0.016)	0.034*
SDMT	-0.469 (-0.819, -0.119)	0.009*	-0.233 (-0.413, -0.053)	0.012*
TMT	1.632 (-0.189, 3.452)	0.078		
SCWT	1.022 (0.138, 1.905)	0.024*	0.251 (0.073, 0.429)	0.006*
VFT	-0.173 (-0.387, 0.041)	0.111		

All linear regression models were adjusted for sex, age, smoking, drinking, hypertension, diabetes, HDL, LDL, HbA1c, and education level

* *P* < 0.05

## Discussion

Cognitive impairment is an age-related issue, and vascular factors (O’Brien & Thomas, [29]) especially CSVD closely relates to it (Pantoni, [30]; Yakushiji et al., [39]). Previous research has demonstrated that age-related alternations of brain venules are involved in CSVD onset (Fulop et al., [10]; Molnár et al., [26]), while studies investigating the relation between brain venules and cognitive dysfunction are insufficient. This paper found that DMV score was independently related to MCI (OR = 1.162, 95%CI= (1.043, 1.295), *P* = 0.007). DMV score also revealed a high diagnostic value for predicting MCI (AUC = 0.822, 95%CI= [0.734, 0.911], *P* < 0.001) when adjusting for confounding factors (Supplementary Fig. 3).

Decreased DMV visibility correlates with worse cognitive performance, and the underlying mechanism may be complicated. Though previous studies have discovered that brain lesions such as WMH, LI, EPVS and CMB were responsible for cognitive impairment (Benisty et al., [1]; Chen et al., [4]; Ding, Wu, Zhou, & Zheng, [7]; Gyanwali et al., [16]; Hachinski, Potter, & Merskey, [17]; Hartmann, Hyacinth, Liao, & Shih, [19]; Jokinen et al., [20]; Longstreth et al., [25]; Moody, Brown, Challa, & Anderson, [27]). Our study did not find any statistical difference in these neuroimaging biomarkers between the MCI group and the control group. We assume that this may due to the slight severity of brain lesions. Brain lesions due to small vessel alternations may be more sensitive for severe cognitive impairment (Hachinski et al., [17]; Wang et al., [37]). Besides, our study chose MoCA rather than MMSE as the criteria for MCI definition. MoCA is more sensitive for detecting deterioration of the message processing function than MMSE, which may also account for the different outcomes of our studies and other research. In the meanwhile, the changes in small veins are ahead of brain lesions. As early as 1995, Moody (Moody et al., [27]) found that with the deposit of collagen in the periventricular venous wall, the venous lumen grows narrow and occlusive, resulting in brain hypoperfusion, brain-blood barrier (BBB) damage, and venous drainage of periventricular white matter blockage. Age-related pathological alternation of venous valves and jugular reflux will cause overall elevation of brain venous system pressure. It will induce the rupture of brain small veins under certain circumstances and the resulting CMBs (Fulop et al., [10]; Gyanwali et al., [16]). Venous collagen deposit and tortuosity also contribute to LI as they do on small arteries (Hartmann et al., [19]). Therefore, it is reasonable to speculate that compared with neuroimaging markers, the DMV score may be a more useful neuroimaging marker to detect early cognitive degeneration.

This study also discovered significant correlation between DMV score and the MMSE (*P* = 0.040), MoCA (*P* = 0.003), AVLT short-term delay (*P* = 0.022), AVLT long-term delay (*P* = 0.020), SDMT (*P* = 0.023), SCWT (*P* = 0.011), TMT (*P* = 0.039) and VFT (*P* = 0.032). DMV score also had a negative association with MMSE (*P* = 0.012), MoCA (*P* = 0.010), AVLT short-time delayed recall (*P* = 0.040), AVLT long-time delayed recall (*P* = 0.034), SDMT (*P* = 0.012) and had a positive association with SCWT (*P* = 0.006). It revealed that DMV may correlate with not only overall cognition, but memory, attention, and executive function as well.

Previous studies have reported that as the age grows, people are more likely to suffer from cognitive impairment for mixed reasons rather than one certain cause (O’Brien & Thomas, [29]), Compared with AD, VCI is more likely to impact frontostriatal circuits and causes attention and executive function damage (O’Brien & Thomas, [29]), but the latest studies suggest that CSVD affect all cognitive domains beyond attention and executive function (de Havenon et al. [5]). Though previous studies investigating alternations of venules and executive function and memory are infrequent, brain lesions are intimately related to memory and executive function. A study based on 442 non-dementia CSVD cases found that the severity and distribution of WMH would impact executive function (Camerino et al., [2]; Ding et al., [7]). WMH between the hippocampus and posterior cingulate cortex was responsible for worse performance in memory (Chen et al., [4]; Jokinen et al., [20]), which could also be mediated by brain network changes (Du et al., [8]). One study demonstrated that the distribution of LI is associated with cognitive dysfunction (Benisty et al., [1]). while another research found no relation between LI and cognitive impairment (Gyanwali et al., [16]). The contradiction may be attributed to racial differences. It is intriguingly that though close correlations between DMV score and deterioration of various cognitive domains were found in our study, but only DWMH was found to correlate with VFT (*P* = 0.032). In other words, DMV score is related to degeneration of cognitive domains independently of brain lesions. Some research found that changes in structural and functional connectivity of brain networks may account for the brain lesion-induced cognitive impairment (Lawrence, Chung, Morris, Markus, & Barrick, [23]; Ter Telgte et al., [36]). Cognitive impairment in CSVD patients is impacted by the decreasing communication efficiency (Lawrence et al., [23]; Ter Telgte et al., [36]), and the decrease in overall network efficiency mediates the transformation from MCI to dementia (Lawrence et al., [24]). Structural connectivity damage of the inner network plays a more significant role compared with that of the outer layer in WMH-mediated executive dysfunction (Reijmer et al., [33]). Besides, brain network functional connectivity has a tight correlation with cognition. The frontoparietal control network relates to executive function (Shaw, Schultz, Sperling, & Hedden, [35]), which may be ascribed to the destruction of cholinergic conductive tracts and frontosubcortical circuits (Dey, Stamenova, Turner, Black, & Levine, [6]). Therefore, DMV score and relevant executive dysfunction may also be mediated by the alternations of brain network damage, which should be verified by further studies.

This study has several limitations. It is a cross-sectional study and we should collect more data to further investigate the causative relation between DMV and cognition. Besides, as we did not evaluate the alternations of brain connectivity, we cannot prove brain connectivity meditates DMV changes and cognitive impairment. Thus further validations are necessary. Third, we performed a semi-quantitative rating for the DMV score, while the quantitative assessment of DMV will be more objective and accurate.

## Conclusion

DMV score independently related to MCI and correlates with overall cognition, memory, attention, and executive function in outpatients.

## Supplementary Information

**Additional file 1: Supplementary Table 1. **Neuroimaging markersfor small vessel disease of included participants.**Supplementary Figure 1.** DMV score A-B: DMV is scaled as 0, consecutiveveins and even signals on SWI sequence; C-D: DMV is scaled as 1, consecutive veins but one or more veins present uneven signals on SWI sequence; E-F: DMV isscaled as 2, one or more discontinuous veins and dotted hypointensity on SWI sequence; G-H: DMV is scaled as 3, no visible consecutive vein on SWI sequence. **Supplementary Figure 2.** Heatmap for Spearman’s correlation between neuroimaging markers and cognition. **Supplementary Figure 3.** ROC curve of adjusted DMV score for MCI. After adjusting for sex, smoking, HDL, HbA1c and education level, DMV score revealed a high diagnostic value for predicting MCI (AUC=0.822, 95% CI= [0.734, 0.911], *P*<0.001), with the sensitivity for MCI is 0.877, the specificity for MCI is0.656.

## Data Availability

The datasets generated and analyzed during the current study are available from the corresponding author on reasonable request.
