# Supplementary material for: Deep medullary veins: a promising neuroimaging marker for mild cognitive impairment in outpatients
Source: BMC Neurol. 2023 Jan 5;23:3. doi: 10.1186/s12883-022-03037-x (PMC9814341; doi:10.1186/s12883-022-03037-x)
Supplement: Supplementary file 1 — Additional file 1: Supplementary Table 1. Neuroimaging markersfor small vessel disease of included participants.Supplementary Figure 1. DMV score A-B: DMV is scaled as 0, consecutiveveins and even signals on SWI sequence; C-D: DMV is scaled as 1, consecutive veins but one or more veins present uneven signals on SWI sequence; E-F: DMV isscaled as 2, one or more discontinuous veins and dotted hypointensity on SWI sequence; G-H: DMV is scaled as 3, no visible consecutive vein on SWI sequence. Supplementary Figure 2. Heatmap for Spearman’s correlation between neuroimaging markers and cognition. Supplementary Figure 3. ROC curve of adjusted DMV score for MCI. After adjusting for sex, smoking, HDL, HbA1c and education level, DMV score revealed a high diagnostic value for predicting MCI (AUC=0.822, 95% CI= [0.734, 0.911], P<0.001), with the sensitivity for MCI is 0.877, the specificity for MCI is0.656. [file 12883_2022_3037_MOESM1_ESM.pdf]

Supplementary Table 1 Neuroimaging markers for small vessel disease of included participants

Characteristics		Total (N=105)	MCI group (N=73)	Control group (N=32)	P
WMH					
PVH, n,(%)					0.343
	0	32 (30.5)	21 (28.8)	11 (34.4)	
	1	32 (30.5)	26 (35.6)	6 (18.8)	
	2	28 (26.7)	17 (23.3)	11 (34.4)	
	3	13 (12.4)	9 (12.3)	4 (12.5)	
DWMH, n,(%)					0.575
	0	31 (29.5)	22 (30.1)	9 (28.1)	
	1	39 (37.1)	29 (39.7)	10 (31.3)	
	2	22 (21.0)	15 (20.5)	7 (21.9)	
	3	13 (12.4)	7 (9.6)	6 (18.8)	
LI, n,(%)		37 (35.2)	26 (35.6)	11 (34.4)	0.902
EPVS					
EPVS in basal ganglia, n,(%)					0.873
	0	47 (44.8)	31 (42.5)	16 (50.0)	
	1	13 (12.4)	10 (13.7)	3 (9.4)	
	2	41 (39.0)	29 (39.7)	12 (37.5)	

	3	4 (3.8)	3 (4.1)	1 (3.1)	
EPVS in centrum semiovale, n,(%)					0.550
	0	65 (61.9)	43 (58.9)	22 (68.8)	
	1	26 (24.8)	18 (24.7)	8 (25.0)	
	2	8 (7.6)	7 (9.6)	1 (3.1)	
	3	6 (5.7)	5 (6.8)	1 (3.1)	
CMB, n,(%)		37 (35.2)	25 (34.2)	12 (37.5)	0.748

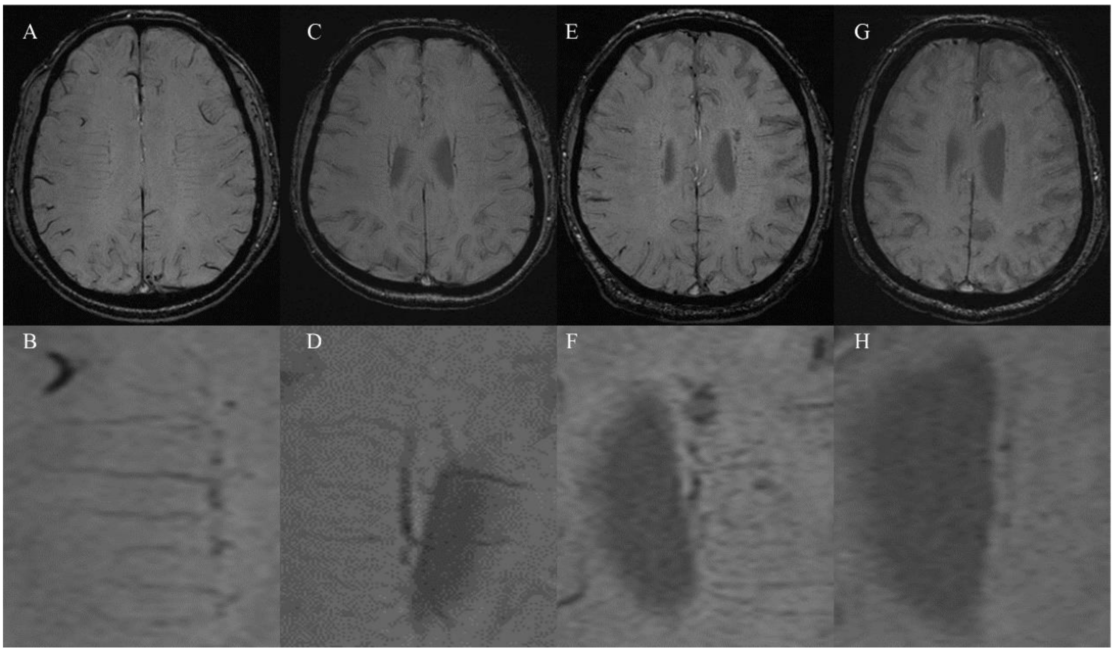

Supplementary Figure 1 DMV score

A-B: DMV is scaled as 0, consecutive veins and even signals on SWI sequence; C-D: DMV is scaled as 1, consecutive veins but one or more veins present uneven signals on SWI sequence; E-F: DMV is scaled as 2, one or more discontinuous veins and dotted hypointensity on SWI sequence; G-H: DMV is scaled as 3, no visible consecutive vein on SWI sequence.

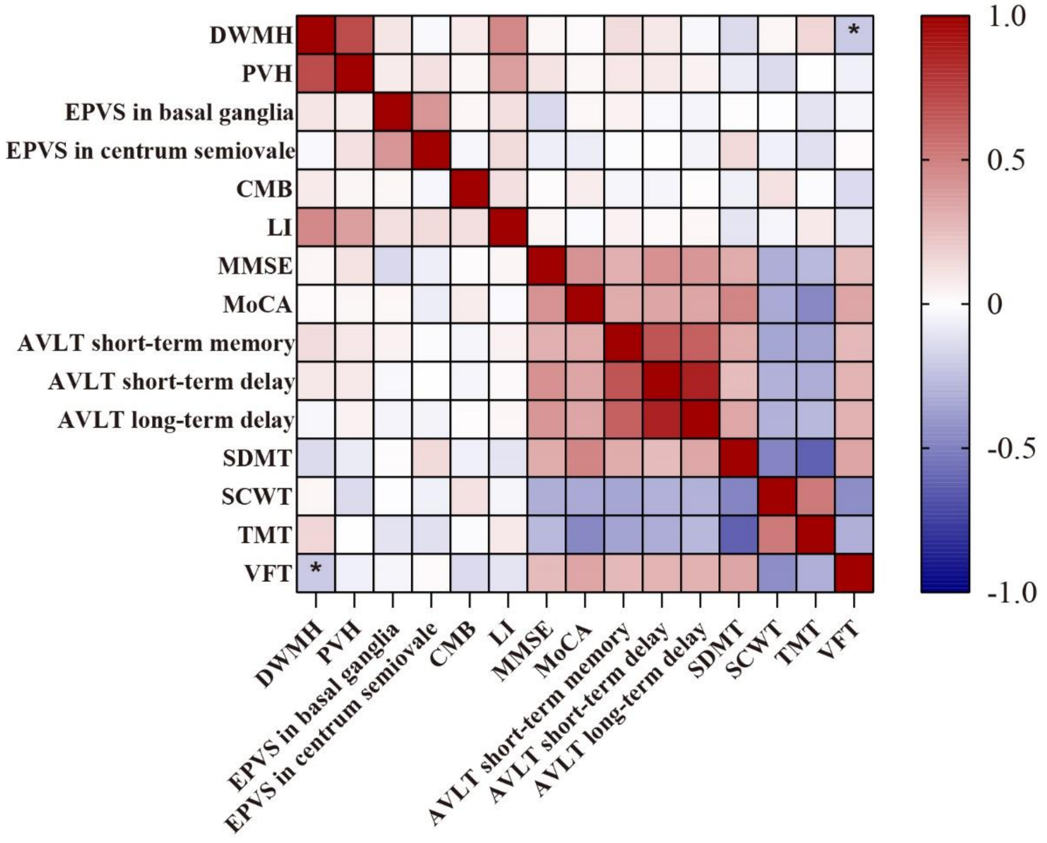

Supplementary Figure 2 Heatmap for Spearman's correlation between neuroimaging markers and cognition

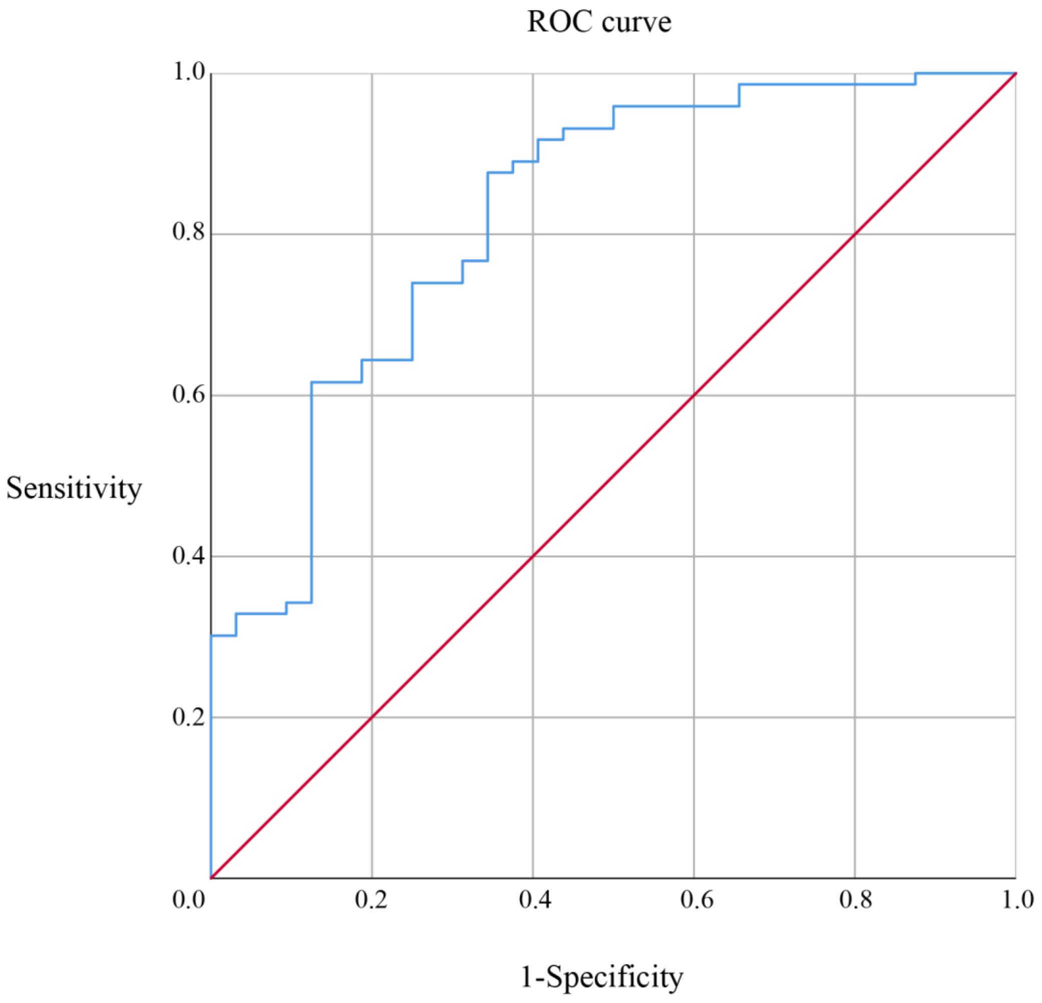

Supplementary Figure 3 ROC curve of adjusted DMV score for MCI

After adjusting for sex, smoking, HDL, HbA1c and education level, DMV score revealed a high diagnostic value for predicting MCI (AUC=0.822, 95%CI= [0.734, 0.911], P<0.001), with the sensitivity for MCI is 0.877, the specificity for MCI is 0.656.
